# Epidemiology of Brucellosis and Genetic Diversity of *Brucella abortus* in Kazakhstan

**DOI:** 10.1371/journal.pone.0167496

**Published:** 2016-12-01

**Authors:** Elena Shevtsova, Alexandr Shevtsov, Kasim Mukanov, Maxim Filipenko, Dinara Kamalova, Igor Sytnik, Marat Syzdykov, Andrey Kuznetsov, Assel Akhmetova, Mira Zharova, Talgat Karibaev, Pavel Tarlykov, Erlan Ramanculov

**Affiliations:** 1 National Center for Biotechnology, Astana, Kazakhstan; 2 Institute of Chemical Biology and Fundamental Medicine, Novosibirsk, Russian Federation; 3 Novosibirsk State University, Novosibirsk, Russian Federation; 4 National Reference Center for Veterinary, Astana, Kazakhstan; 5 Kazakh Scientific Center of Quarantine and Zoonotic Diseases named by Masgut Aykimbayev, Almaty, Kazakhstan; 6 School of Science and Technology Nazarbayev University, Astana, Kazakhstan; Institut National de la Recherche Agronomique, FRANCE

## Abstract

Brucellosis is a major zoonotic infection in Kazakhstan. However, there is limited data on its incidence in humans and animals, and the genetic diversity of prevalent strains is virtually unstudied. Additionally, there is no detailed overview of Kazakhstan brucellosis control and eradication programs. Here, we analyzed brucellosis epidemiological data, and assessed the effectiveness of eradication strategies employed over the past 70 years to counteract this infection. We also conducted multiple loci variable-number tandem repeat analysis (MLVA) of *Brucella abortus* strains found in Kazakhstan. We analyzed official data on the incidence of animal brucellosis in Kazakhstan. The records span more than 70 years of anti-brucellosis campaigns, and contain a brief description of the applied control strategies, their effectiveness, and their impact on the incidence in humans. The MLVA-16 method was used to type 94 strains of *B*. *abortus* and serial passages of *B*. *abortus* 82, a strain used in vaccines. MLVA-8 and MLVA-11 analyses clustered strains into a total of four and seven genotypes, respectively; it is the first time that four of these genotypes have been described. MLVA-16 analysis divided strains into 28 distinct genotypes having genetic similarity coefficient that varies from 60 to100% and a Hunter & Gaston diversity index of 0.871. MST analysis reconstruction revealed clustering into "Kazakhstani-Chinese (Central Asian)", "European" and "American" lines. Detection of multiple genotypes in a single outbreak confirms that poorly controlled trade of livestock plays a crucial role in the spread of infection. Notably, the MLVA-16 profile of the *B*. *abortus* 82 strain was unique and did not change during 33 serial passages. MLVA genotyping may thus be useful for epidemiological monitoring of brucellosis, and for tracking the source(s) of infection. We suggest that countrywide application of MLVA genotyping would improve the control of brucellosis in Kazakhstan.

## Introduction

The incidence of brucellosis reaches as much as 200 cases per 100,000 of the population in some regions of the world; besides, the infection has become endemic in many countries [1]. In addition, brucellosis surveillance data often underestimates the true incidence owing to diagnostic errors, the diversity of the clinical cases, and concealed official data [2, 3] The majority of brucellosis cases are registered in Mediterranean countries, South and Central America, Africa, Asia, Indian subcontinent, Eastern Europe, and the Middle East [4, 5]. Successful implementation of an animal vaccination program, in addition to testing and slaughter of animals which are suspected of (or test positive for) the disease has conferred "officially bovine brucellosis free" and/or "officially ovine and caprine (*Brucella melitensis*) free" status on a number of countries [6]. Control in animals is critical for prevention of spread to humans; indeed, the majority of human cases in brucellosis-free regions are due to import by people who have traveled to endemic regions, as they may have had contact with wild animals or with imported products [7–10]. Local eradication of brucellosis, followed by reduction of control, may lead to dramatic consequences, as evidenced by re-emergence in Pacific Island Countries [11], France [12], Malta, and Oman [13]. Continued surveillance of brucellosis in disease-free area is thus important to prevent re-emergence of the zoonosis [14].

Molecular characterization of circulating strains of *Brucella* is critical for elimination of repeated outbreaks, tracking infection spread, and informing selection of anti-brucellosis strategies [15, 16]. Multiple-Locus Variable-number tandem-repeat Analysis (MLVA) is a well-suited technique for genotyping of *Brucella* [17–19]. Genotyping of *Brucella* strains enables differentiation between natural and deliberate outbreaks of disease. This is important because the bacterium is highly contagious in both humans and animals, and the organism can be readily aerosolized and used as a weapon by bioterrorists; furthermore, the infection is associated with non-specific symptoms [20–23]. Existing MLVA and Multilocus sequence typing (MLST) databases for typing of bacteria have greatly facilitated global epidemiologic studies, and international cooperation aimed at limiting spread of the disease [24–26].

Kazakhstan, a former Soviet republic located in central Eurasia, is in the top twenty five states with the highest incidence of brucellosis [5]. The country has implemented a number of brucellosis eradication programs over the past 80 years; however, epidemiological data over this period is scarce. Therefore, it is hard to infer effectiveness of these activities, and how the prevalence of brucellosis in animals and humans has changed over time. Finally, there is no accurate and up to date record of the precise *Brucella* genotypes in circulation.

Our previous study revealed a low genetic diversity among 124 strains of *B*. *abortus* isolated from animals found in two regions of Kazakhstan. Specifically, the index of genetic diversity (HGDI) for MLVA-16 was 0.491 [27]. The discriminatory ability of this study was not high enough for epidemiological monitoring, possibly due to the limited sampling area. To address this issue, we expanded the sampling area and timeline by including samples from repositories dating back to 1948. Secondary goals of the current work were to analyze *Brucella* epidemiology in Kazakhstan, and to evaluate the efficacy of current brucellosis eradication programs.

## Materials and Methods

### Bacterial strains and DNA preparation

A total of 89 *B*. *abortus* strains were isolated from different veterinary samples (including blood, synovia, and aborted fetuses). Twenty-five strains have been isolated between 1943 and 1985 and stored in a repository in a cryopreserved or freeze-dried state with periodic changes of the liquid selective media. *Brucella abortus* strain 544 has been stored under the same conditions since 1964, and was used to evaluate the genetic stability upon storage. The other 64 strains were collected from seropositive animals detected during routine testing for brucellosis in 2015. Strains were cultivated on Brucella Agar with Hemin and Vitamin K (HiMedia Laboratories) supplemented with 10% horse serum (Sigma-Aldrich) and Brucella Selective Supplement (HiMedia Laboratories). The strains were identified as *Brucella* species by the means of classical identification protocol based on growth inhibition by thionine and basic fuchsine, in addition to measurement of cytochrome oxidase, catalase, and urease activity according to the published protocol [28]. The strains collected between 2007 and 2012 were preserved in cryoprotectant as described elsewhere [29]. Strains collected in 2013 and later were processed without prior cryopreservation. The National Reference Center for Veterinary Medicine of the Republic of Kazakhstan (NRCVM) was a sample source and main state repository of the selected strains. *B*. *abortus* RB51 and *B*. *abortus* S19 were used as reference strains to verify fragment sizes by comparison to the MLVA database. The commercial vaccine strain *B*. *abortus* 82, which is manufactured in Russia, was used to evaluate circulation of vaccine strains. The provided samples were subjected to DNA isolation using a DNeasy Blood & Tissue Kit (Qiagen), according to the instructions provided by manufacture. Sequencing of *16S rRNA* gene was performed with universal primers8F (5′-AGAGTTTGATCCTGGCTCAG-3′) and 806R (5′-GGACTACCAGGGTATCTAAT-3′) Possible bacterial contamination was also examined by sequencing [30]. Bruce-ladder PCR was used for species-level identification [31].

### MLVA genotyping

The selected MLVA markers were previously reported by Le Flèche et al. [32], and refined by Al Dahouk et al. [17]. Multiplex PCR and capillary electrophoresis (CE) were carried out as described by Garofolo et al. with minor modifications [27, 33]. The sixteen pairs of primers were separated into three panels. Panel 1, also known as MLVA-8, consists of8 loci (Bruce 06, Bruce 08, Bruce 11, Bruce 12, Bruce 42, Bruce 43, Bruce 45, and Bruce 55). Panel 2A has three additional loci(Bruce 18, Bruce 19, and Bruce 21), and panel 2B has five extra loci (Bruce 04, Bruce 07, Bruce 09, Bruce 16, and Bruce 30). Combination of MLVA-8 and 2A loci results in the MLVA-11 panel, while combination of all three panels (16 loci) results in MLVA-16. VNTR fragment sizes were automatically calculated by GeneMapper software 4.1 (Applied Biosystems). Allele calling of multiplexed products was conducted as described previously [33]. The resulted data were processed with BioNumerics 7.5 software (Applied Maths,). MLVA-16 profiles of five additional strains were included from our previous study (“key”: a_1, a_18, a_82, a_112 and a_121) [27], All of them were isolated from cattle between 2007 and 2013 and found to have different genotypes. Cluster analysis was based on the categorical coefficient and unweighted pair group method using arithmetic averages (UPGMA). Standard minimum spanning trees (MSTs) were generated using categorical coefficients together with the single and double locus variance priority rules.

Recent discovery of rare alleles has changed the nomenclature of the locus Bruce 19 and thus introduced ambiguity; therefore, it was excluded from computation of the MST trees. HGDI was calculated for polymorphisms at each locus via V-DICE tool available at the HPA website (http://www.hpa-bioinformatics.org.uk/cgi-bin/DICI/DICI.pl) [34]. The genotypes were identified by comparison with the May 2016 version of the MLVA database (MLVAbank, http://microbesgenotyping.i2bc.paris-saclay.fr).

### Epidemiology data

A literature search was carried out in March 2016 by means of three electronic databases: MEDLINE, Google Scholar, and Web of Science, using different combinations of the following keywords in titles and/or abstracts: “brucell”, “livestock” “cow* OR cattle OR ruminant* OR sheep OR goat* OR farm* OR smallholding* OR collective*”. These keywords were combined with the name of the country: “Kazakh*”.

Article title and abstracts were examined for compliance with the search criteria. Full texts of articles were obtained if they met the selection criteria. Published articles on the epidemiology of brucellosis in Kazakhstan in Russian and Kazakh were also included in the search. Official statistics on the brucellosis incidence in livestock were taken from the annual reports issued by "Republican Veterinary Laboratory", as well as reports issued by the "Ministry of Agriculture of the Kazakh SSR".

## Results

### Brucellosis incidence and eradication strategies in Kazakhstan

**Fig 1:**
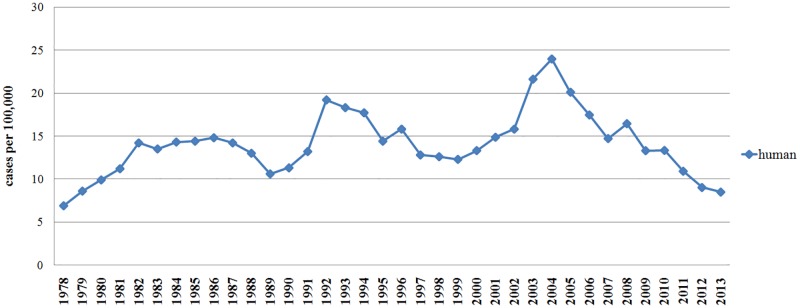
Incidence of human brucellosis. Time in years is on the x-axis, incidence per 100,000 population is on the y-axis.

The first case of human brucellosis was recorded in 1932 in the southern regions of Kazakhstan. Two years later, brucellosis was reported in central Kazakhstan. Medical expedition to remote areas with semi-nomadic pastoralism that took place in 1937 revealed a high incidence of brucellosis among the local population, which indicates that there was a continuous circulation of *Brucella* in Kazakhstan by this time [35]. However, it is not possible to obtain a complete picture of *Brucella* dissemination during this period owing to non-systematic record keeping of brucellosis morbidity at the time. Between 1978 and 1990, the incidence of human brucellosis in the country ranged from 6.9 to 14.8 cases per 100,000 population, with the sharpest rise occurring between 1982 and 1988 (Fig 1).

**Fig 2:**
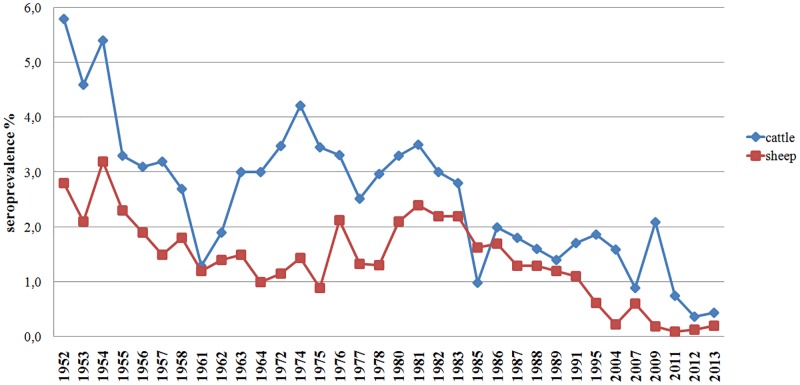
Incidence of animal brucellosis. Time in years is on the x-axis, seroprevalence (%) is on the y-axis.

The first cases of brucellosis in animals were registered in Kazakhstan in 1930 [36], while planned diagnostic testing for brucellosis was introduced in 1932. The first systematic data were presented in the annual reports of the Republican veterinary laboratory. The first available issues date back to 1952 and indicate high infection rates in cattle and sheep of 5.8 and 2.8%, respectively (Fig 2).

Fluctuations in the incidence of animal brucellosis in the Soviet era can be attributed to the timing of various strategies that were designed to achieve brucellosis eradication. Extensive vaccination of cattle with the strain *B*. *abortus* S19 began in 1955 and provided 70% cattle coverage until 1971. According to epidemiological data, there was a considerable decline in morbidity, which resulted in a reduced number of abortions during this period. Nevertheless, the level of detection of seropositive animals remained high owing to an inability to discriminate vaccinated and infected animals, and prolonged preservation of antibrucellar antibody titer in the vaccinated animals. For this reason, vaccination with strain *B*. *abortus* S19 was discontinued in 1971 in many regions of the country. This had adverse effects on the epizootic situation in the country, leading to an increase in seropositive animals that peaked at 4.2% in 1974. Moreover, a deteriorating situation occurred in the northern regions, where there was a high concentration of animals and a long stall period [37, 38]. Since the mid-70s, *B*. *abortus* strain 82 has been used to vaccinate all groups of cattle along with state-wide test and slaughter program, which led to a gradual decrease of incidence in herds [39, 40], reaching its minimum (1.4%) in 1989. Hypothetically, continuation of this combined strategy would eradicate brucellosis from cattle.

However, after the fall of the Soviet Union and its economy in 1991, agriculture regulations became outdated. Large collective farms were de-nationalized, which led to a sharp decline in the number of livestock and the formation of many small private farms [41]. The previous system of veterinary control thus became ineffective. In addition, the difficult economic situation contributed to the deterioration of the control of many infectious diseases, including brucellosis [42]. Officially, the vaccination program continued until 2006; however, the program virtually collapsed in 1992 due to lack of funding, and shutdown of industrial complexes for vaccine production that led to an increase in vaccine cost [43]. A coincidence of the circumstances thus led to an increase in the number of seropositive cattle to 1.9% in 1995.

Official data associated with this time period are best viewed as highly inconsistent, with widely fluctuating infection rates. For example, in 2007 only 0.6% of 440,000 animals tested were diagnosed as seropositive. Since 2007, vaccinations were completely cancelled and replaced with a new strategy that included total diagnostic coverage of animals using an enzyme-linked immunosorbent assay (ELISA) and subsequent slaughter of seropositive animals. Application of ELISA has dramatically increased the detection of seropositive cattle. For example, 2.1% of 5.5 million samples were seropositive in 2009. Despite the subsequent decline in the number of seropositive animals, voluntary vaccination of cattle was incorporated into the brucellosis eradication program in 2014. Voluntary vaccination using cattle vaccines registered in Kazakhstan (*B*. *abortus* S19, *B*. *abortus* 82, *B*. *abortus* RB-51) and sheep vaccine (*B*. *melitensis* Rev1) was permitted. Selection of the vaccine is now the prerogative of the veterinarian and cattle owner, who must notify the State Veterinary Service of their activities. This introduces an additional burden on the veterinary surveillance system and diagnostic laboratories, both in terms of differentiation of vaccinated and infected animals, as well as the isolation of bacterial cultures.

Test-and-slaughter of seropositive animals was the main strategy for control of brucellosis in sheep until the mid-1950s. At this time, sheep population coverage had not reached 50%. This tactic has therefore had no significant impact on the epizootic situation in the country. From 1956 to 1974, a brucellosis vaccination campaign (with *B*. *abortus* S19 vaccine) was carried out in sheep of any age with a preliminary test-and-slaughter of seropositive cattle. The campaign led to a 50% reduction in the incidence of sheep brucellosis between 1956 and 1975. Since 1974, young sheep began to receive the Rev1 vaccine, while the adult population continued to be vaccinated with *B*. *abortus* S19 until 1979. Subsequently, single immunization of young animals with Rev1 vaccine was adopted. This coincided with a difficult epidemiological situation when groups of animals of different age were held together. As a result, the strategy proved to be ineffective, which led to an increase in the brucellosis incidence between 1980 and 1983 that equaled the rates observed in 1953 (Fig 2). In addition, the incidence of human brucellosis was adversely affected, reaching 14.8 cases per 100,000 individuals. Since 1983, obligatory vaccination of adult sheep with *B*. *abortus* S19 has been introduced. In the late 1980s, Rev 1 vaccine was recommended for immunization of young animals and ewes every two years. This approach led to a 50% reduction in the number of seropositive animals between 1983 and 1989.

Since 1991, brucellosis incidence in sheep has declined steadily, and appears to have stabilized at 0.1–0.3% by 2011. Changes in veterinary regulations and control strategy with full coverage testing of livestock, followed by slaughter of seropositive animals have not significantly affected the level of infection. In contrast to the decline in sheep, the incidence in humans steadily increased from 11.3 per 100,000 individuals in 1990 to 19.2 per 100,000 in 1992, and reached a peak (23.95/100,000 people) in 2004 [43]. Afterwards, the incidence of human brucellosis declined steadily, reaching an average of 8.5 cases per 100,000 in 2013. However, the incidence varies in different regions of the country, with some areas of southern Kazakhstan reaching up to 150 cases per 100,000 people [44]. Hypothetically, true incidence may be underestimated, as the current criteria for infection is very stringent: *Brucella* growth in culture medium must be observed in order to identify the subject as positive for the bacterium [43].

### Genotyping of *Brucella abortus* strains using the MLVA16 assay

**Fig 3:**
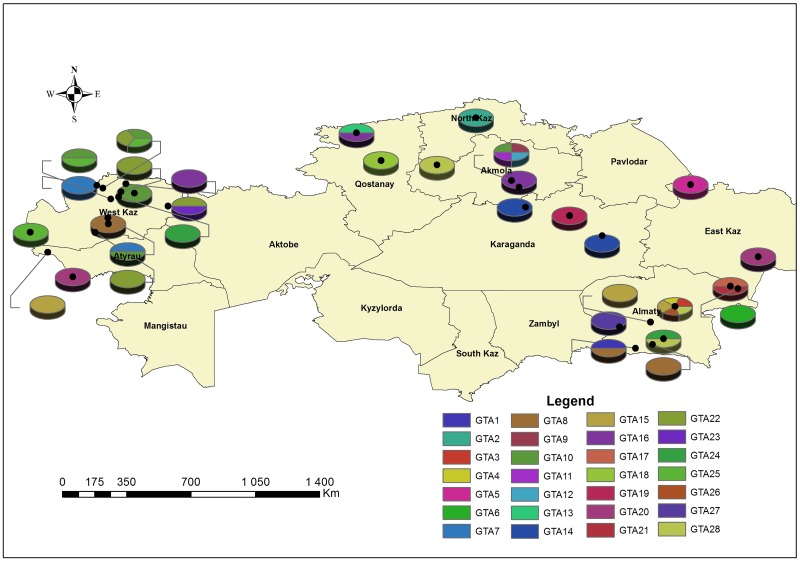
Geographical representation of *Brucella abortus* sample collection sites.

*B*. *abortus* isolates have been collected from 8 regions representing 27 villages; however, not every isolate collected before 1970 had its precise geographic collection point specified (Fig 3, S1 Table). Ninety-three strains were isolated from cattle, and one from camel.

**Fig 4:**
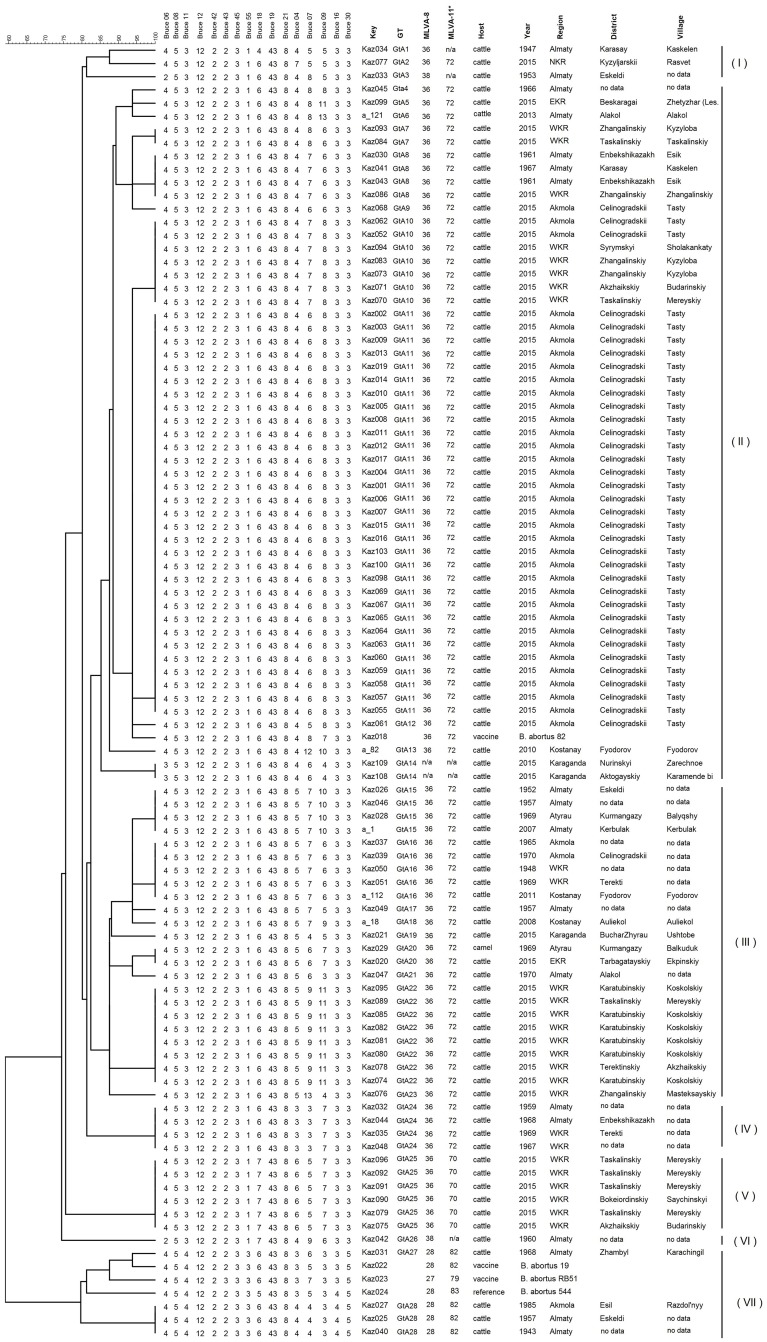
Cluster analysis for 94 strains of *Brucella abortus* based on the MLVA-16 dataset. In the columns, the following data for strains are indicated: Key, serial number for the strain in the MLVA bank; GT, genotype MLVA16 in this study; MLVA-8 and MLVA-11, genotype numbers associated with the genotypes corresponding to each strain in the database; region, geographic region (NKR, North Kazakhstan Region; EKR, East Kazakhstan Region; WKR, West Kazakhstan Region); host, animal host; year, year of isolation.

The strains from the sample collection were assigned to the genus *Brucella* based on the nucleotide sequences analysis of the 16S rRNA gene. There were no overlapping peaks present in the sequencing chromatograms. Discrimination of *Brucella* species was based on the Bruce-ladder PCR. All *Brucella* samples were identified as *Brucella abortus*. VNTR analysis of 16 loci was carried out on all samples. The MLVA profiles from the current work have been uploaded to an international *Brucella* MLVA database (http://microbesgenotyping.i2bc.paris-saclay.fr) and Figshare repository (https://dx.doi.org/10.6084/m9.figshare.4244477.v1). The genotyping results were validated by matching the profile of the three reference strains *B*. *abortus* S19, *B*. *abortus* RB51, and *B*. *abortus* 544. The MLVA-16 profiles of the reference strains were identical to previously published profiles in the MLVA database. Limited diversity was observed with MLVA-8 and MLVA-11, Table 1 (HGDI 0.162 and 0.290, respectively). Analysis of the panel A loci (MLVA-8) revealed four genotypes, with genotype 36 being the most common among the strains (91%, n = 86). Four strains were found to belong to genotype 28, two strains to genotype 38, and two strains to genotype 167,which has been detected earlier in Brazil [45]. Three loci that contributed to discrimination in the set of MLVA-8 were Bruce 06 (HGDI of 0.083), Bruce 11, and Bruce 55 with an HGDI of 0.082. MLVA-11 analysis of 94 *B*. *abortus* strains resulted in seven different genotypes. The most common genotype, 72, represented 84% of the strains (n = 79), followed by genotype 70 (6.4%, n = 6), and genotype 82 (4.3%, n = 4). The other five strains were classified into four different MLVA-11 genotypes that were not found in the MLVAbank. These genotypes have been submitted to MLVAbank and recently assigned as genotypes 338 to 341. The Panel 2A locus, Bruce 18, did not have any significant discriminatory capacity, and was associated with an HGDI of 0.159. MLVA-16 analysis of the strains resulted in twenty-eight distinct genotypes with genetic similarity coefficient that varies from 60% to100% (Fig 4).

**Table 1:** Allelic types and HGDI of *B*. *abortus* strains for 16 loci in this study.

Locus	*B*. *abortus*			
	Diversity Index	Confidence Interval	K^a^	max (pi)^b^
**Panel 1**				
**Bruce 06**	0.083	0.007–0.160	3	0.957
**Bruce 08**	0.000	0.000–0.074	1	1.000
**Bruce 11**	0.082	0.008–0.157	2	0.957
**Bruce 12**	0.000	0.000–0.074	1	1.000
**Bruce 42**	0.000	0.000–0.074	1	1.000
**Bruce 43**	0.000	0.000–0.074	1	1.000
**Bruce 45**	0.000	0.000–0.074	1	1.000
**Bruce 55**	0.082	0.008–0.157	2	0.957
**Panel 2A**				
**Bruce 18**	0.159	0.063–0.255	3	0.915
**Bruce19**	0.000	0.000–0.074	1	1.000
**Bruce 21**	0.000	0.000–0.074	1	1.000
**Panel 2B**				
**Bruce 04**	0.553	0.465–0.640	5	0.617
**Bruce 07**	0.764	0.705–0.823	9	0.404
**Bruce 09**	0.779	0.714–0.844	10	0.415
**Bruce 16**	0.062	0.000–0.129	2	0.968
**Bruce 30**	0.082	0.008–0.157	2	0.957
**MLVA-8**	0.162	0.062–0.262	4	0.915
**MLVA-11**	0.290	0.172–0.408	7	0.840
**MLVA-16**	0.871	0.816–0.926	28	0.330

^a^K = Number of alleles observed at this locus in this sample set.

^b^max (pi) = Fraction of samples that have the most frequent repeat number in this locus (range 0.0–1.0)

Sixteen of them were represented by single strains, and the associated dendrogram revealed seven main clades. The first consists of three strains classified as genotypes GtA1-GtA3, while the second one is the most abundant with 52 strains (genotypes GtA4-GtA14), including a vaccine strain of *Brucella abortus* 82. The third clade consists of 24 strains and 9 genotypes (GtA15-GtA23). The fourth, fifth, and sixth clades consisted of genotypes GtA24-GtA26 clustered together. The seventh clade was comprised of *B*. *abortus* RB51, *B*. *abortus* S19, and *B*. *abortus* 544 reference strains, as well as four strains with genotypes GtA27, GtA28. The total HGDI for all 16 loci was 0.871. All Panel 2B’s loci had high discriminatory potential, with the highest variability observed at loci Bruce 09 (HGDI 0.779), Bruce 07 (HGDI 0.764) and Bruce 04 (0.553). The Bruce 30 and Bruce 16 loci had only two alleles with HGDI 0.082 and 0.062, respectively.

**Fig 5:**
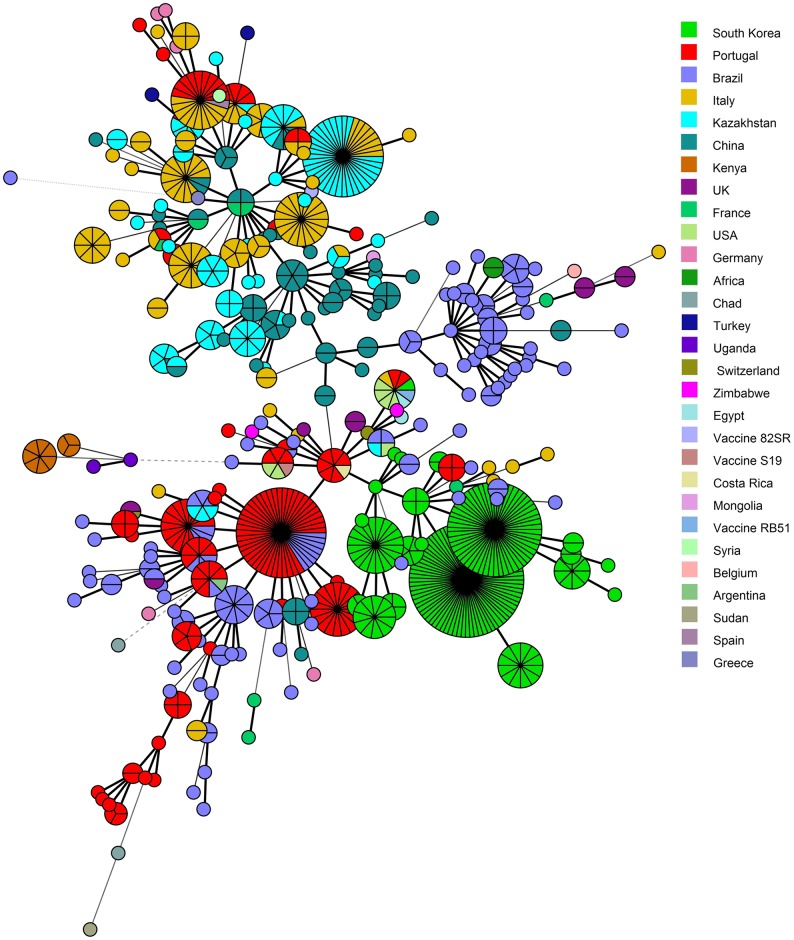
Minimum spanning tree for 94 local isolates of *Brucella abortus* using MLVA-15 data from 769 profiles of *B*. *abortus*.

The generated MST was based on 94 MLVA-15 profiles of the *B*. *abortus* strains from the current study, and 769 strains of *B*. *abortus* from the MLVA bank (Fig 5). MST analysis reconstruction revealed clustering of ninety strains (earlier classified as I–VI clades) with the strains prevalently circulating in countries of Europe and Asia, including Italy, Germany, France, some areas of Portugal, and China. Interestingly, dominant strains in China are arranged in the lower part of the cluster, forming a single group with the clade III strains. European strains are arranged in the upper part of the cluster with clades I, II, and IV–VI strains. The MLVA profile of clade VII strains was identical to that of strains from Brazil and the United States; these latter strains clustered with those from America, Korea, and Portugal. Eighteen genotypes in this study (GaA1-GtA7, GtA12-GtA14, GtA16, GtA17 and GtA21-GtA26) representing 39 (41.5%) strains had unique profiles that have no match with MLVAbank profiles. MST analysis revealed clustering of the other 55 (58.5%) strains with genotypes circulating in Italy (GtA9-GtA11 and GtA20) [46], Portugal (GtA8 and GtA9) [47], and China (GtA10, GtA15, GtA18, GtA19) [48].

It is evident that a number of genotypes have been circulating in Kazakhstan for a long time. For example, genotype GtA8 was in circulation in the Almaty region in the 1960s, and was detected in the West Kazakhstan region as recently as 2015 (Figs 3 and 4, S1 Table). Genotype GtA15 was identified in the Almaty region in the 1950s, and emerged again in 2007. In addition, the genotype was detected in Atyrau region in 1969. Genotype GtA16 has been found in three areas between 1948 and 2011. Interestingly, strains belonging to the “American” lineage with MLVA-8 genotype 28 were not detected after 1985.

In some cases, several genotypes could be linked to specific places. For example, GtA25, GtA7, and GtA22 were isolated strictly in the West Kazakhstan region, while two strains with the new MLVA-8 genotype were isolated in two unrelated outbreaks in Karaganda region. Thirty-one strains with genotype GtA11 were identified in a cattle breeding farm at the Tasty village in the Akmola region during the disease outbreak (June-October 2015). This outbreak also revealed 4 strains belonging to three different genotypes (Gta12, Gta9, and Gta10).

The MLVA-16 profile of vaccine strain *B*. *abortus* 82 was not 100% identical to the strains from this study, or to strains deposited in MLVAbank prior to May 2016. Genetically related strains that were isolated in Italy, China, and in our current study differed from the vaccine strain in Bruce 07 and Bruce 09 loci by 1–3 repeats. The loci of two vaccine strains of *B*. *abortus* 82 that we tested were highly stable, as they did not change over 33 consecutive passages in culture. One of these strains was purchased as a vaccine (Kursk Biofactory, Russia), while the second was kept in a depository of NRCVM (unpublished data).

## Discussion

The post-Soviet republics of Central Asia, including Kazakhstan, are considered hyperendemic regions for brucellosis [5]. Brucellosis in humans and animals is highly prevalent in Kazakhstan with the incidence being especially elevated in its southern and south-eastern provinces, where economic role of ruminant livestock is vital. Besides, 62 species of wild mammals in Kazakhstan are estimated to be seropositive for brucellosis [49].

Despite a century-long history of anti-brucellosis campaigns, Kazakhstan has not overcome this public health threat. According to official data, the best results have been achieved in sheep, where brucellosis has almost been eradicated via vaccination of young and adult population. A combination of the test-and-slaughter strategy and vaccination program reduced the incidence of sheep brucellosis to 1% by the beginning of the 1990s, and this most likely contributed to a decrease in the human brucellosis incidence. Over the following twenty years, the incidence of seropositive sheep dropped to 0.1%, which is close to the level of non-specific error [43]. Interestingly, this finding contradicts medical data, which states there have been 19,145 reported human cases of brucellosis over the past 10 years in Kazakhstan, or an average of 1,900 new cases annually (unpublished data). Notably, 99.9% of these cases were caused by *B*. *melitensis*, indicating that sheep were a main source of infection. However, *B*. *abortus* seems to be present as well, especially when the highest seroprevalence rates were reported in animals, [50]. Among the factors that contribute to the human brucellosis burden in the post-Soviet period is the increased number of people employed in sheep breeding. Approximately 7- to 8-fold more people are now involved in sheep breeding when compared to the period in which Kazakhstan was a Soviet republic state. Another reason is that the use of state slaughterhouses has been replaced by home slaughter[43]. The incidence of brucellosis in cattle is not constant, and changes immediately in response to eradication strategies. Currently, there is no single approved strategy to fight brucellosis, as evidenced by the constant changes in strategy formulation. Regardless of this, and of the government’s efforts to detect infected animals, the uncontrolled trade in animals is considered the main source of herd incidence [51, 52]. Therefore, genetic fingerprinting of isolated strains can have a significant impact on outbreak control, and can facilitate the investigation of infection distribution.

In the current study MLVA-16 was used to assess the genetic diversity among strains of *B*. *abortus* collected from different regions of Kazakhstan since 1943. The combined analysis of 16 VNTR markers revealed a high level of genetic diversity (HGDI, 0.871). Attributable to the broader geographical coverage and temporal sampling interval, this is almost twice the value that was obtained in an earlier study [27]. Comparison of MLVA-11 profiles with MLVAbank data revealed four new genotypes. Interestingly, the most common genotype was MLVA-8 genotype 36, which is distributed throughout Eurasia. Previously published data indicate a high genetic identity of *B*. *melitensis* strains from China and Kazakhstan, indicating the relevance of geographical proximity and historical interaction between countries in animal husbandry [53, 54]. Data on genetic diversity of *B*. *abortus* strains reveals "Kazakhstani-Chinese" relationships, as well as the presence of European and several American genotypes. This is likely due to the large-scale import of pedigree cattle that began at the beginning of the last century to improve the native breeds of cattle. Specifically, despite their high resistance to infections and adaptability to nomadic pastoralism, the native Kazak breeds were characterized by low body weight (280–320 kg) and milkability (500–600 kg per lactation) [55]. It is possible that the “American” lineage of *Brucella* was been imported with Hereford cattle from Uruguay or Santa Gertrudis in the United States. Notably, MLVAbank has only three strains with MLVA-11 genotype 70 that were registered in Germany, while our study has uncovered six strains isolated in three localities of the West Kazakhstan region. Interestingly, West Kazakhstan has specialized in the breeding of Simmental cattle since the 1930s when they were imported to from the Ukraine and Russia, and later from Germany and Switzerland [56].

Remarkably, most outbreaks in the current study are characterized by multiple genotypes. For example, the strains isolated in Budarinskiy village (West Kazakhstan) were classified as genotypes GtA10 and GtA25 with three hypervariable 2B panel loci (Bruce04, Bruce07, and Bruce09) and one panel 2A locus (Bruce 18). Another example is an outbreak in Mereyskiy village (West Kazakhstan) represented by three major genotypes GtA10, GtA22 and GtA25 with the same hypervariable loci. Outbreaks in Tasty (Akmola region) were represented by four genotypes (GtA9-GtA12). The high genetic stability of MLVA-16 markers [31, 57] and changes in the number of VNTR loci in panels 2A and 2B suggest several sources of infection. We infer that these data indicate the role that poorly controlled trade of livestock plays in the emergence of outbreaks. MLVA-16 analysis confirmed the distribution of genotype GtA25 in West Kazakhstan. According to the epidemiological data, the villages of Budarinskiy and Saychinskiy received their heifers from Mereyskiy village six and nine months before brucellosis outbreaks, respectively. Genotype GtA25 was isolated exclusively from the livestock purchased in Mereyskiy village, where this genotype had also previously been found. It was not possible to trace the source of the other genotypes.

The unique profile of the vaccine strain *B*. *abortus* 82 and the high stability of the genetic loci within the MLVA-16 panel suggest that MLVA analysis can be used for primary identification of field and vaccine strains. Nevertheless, the search for alternative highly specific discriminatory markers for the vaccine strain is needed, since certain VNTR loci are unstable over multiple passages. Unfortunately, their stability under selective pressure exerted by the host has not been studied [38].

## Conclusion

Although Kazakhstan is an endemic area for brucellosis, the epidemiological data on incidence of the disease in humans and animals is limited. This study represents the first comprehensive report on brucellosis epidemiology in Kazakhstan over the last 70 years. In addition, this study shows that the genetic diversity of *B*. *abortus* in Kazakhstan is greater than previously reported, and emphasizes the importance of extending the geographical and temporal coverage of sampling as widely as possible. Certain genotypes are widely distributed from both temporal and geographical perspectives, while some outbreaks are characterized by strictly defined genotypes. Globalization and poor control of imported animals can significantly affect the distribution of genotypes as evidenced by the circulation of European, Asian, and American lines. We recommend that epidemiological surveillance should be improved, and that MLVA analysis should be introduced into veterinary control measures, as this will allow the tracking of the geographical location of genotypes. Moreover, when compared with routine biotyping, it will permit the differentiation of field and vaccine strains, and will reduce the risk of infection of personnel.

## Supporting Information

S1 Table Characterization of the collected *Brucella abortus* strains including MLVA-16 genotypes. (XLS)
